# Effects of Resting vs. Continuous Blood-Flow Restriction-Training on Strength, Fatigue Resistance, Muscle Thickness, and Perceived Discomfort

**DOI:** 10.3389/fphys.2021.663665

**Published:** 2021-03-30

**Authors:** Carsten Schwiete, Alexander Franz, Christian Roth, Michael Behringer

**Affiliations:** ^1^Department of Sports Medicine and Exercise Physiology, Institute of Sports Sciences, Goethe University Frankfurt, Frankfurt am Main, Germany; ^2^Department of Adult Reconstruction, ATOS Orthoparc Clinic Cologne, Cologne, Germany

**Keywords:** blood-flow restriction, hypertrophy, maximum strength, fatigue resistance, perceived discomfort

## Abstract

**Introduction**: The purpose of this study was to clarify whether blood-flow restriction during resting intervals [resting blood-flow restriction (rBFR)] is comparable to a continuous BFR (cBFR) training regarding its effects on maximum strength, hypertrophy, fatigue resistance, and perceived discomfort.

**Materials and Methods**: Nineteen recreationally trained participants performed four sets (30-15-15-15 repetitions) with 20% 1RM on a 45° leg press twice a week for 6 weeks (cBFR, *n* = 10; rBFR, *n* = 9). Maximum strength, fatigue resistance, muscle thickness, and girth were assessed at three timepoints (pre, mid, and post). Subjective pain and perceived exertion were determined immediately after training at two timepoints (mid and post).

**Results**: Maximum strength (*p* < 0.001), fatigue resistance (*p* < 0.001), muscle thickness (*p* < 0.001), and girth (*p* = 0.008) increased in both groups over time with no differences between groups (*p* > 0.05). During the intervention, the rBFR group exposed significantly lower perceived pain and exertion values compared to cBFR (*p* < 0.05).

**Discussion**: Resting blood-flow restriction training led to similar gains in strength, fatigue resistance, and muscle hypertrophy as cBFR training while provoking less discomfort and perceived exertion in participants. In summary, rBFR training could provide a meaningful alternative to cBFR as this study showed similar functional and structural changes as well as less discomfort.

## Introduction

It has long been assumed that high mechanical stress is required to achieve improvements in muscle mass and strength. In this context, the American College of Sports Medicine (ACSM) recommends exercising with at least 65% of the one repetition maximum (1RM) to induce hypertrophy in the skeletal muscle (American College of Sports Medicine, 2009). However, there has been an increasing number of studies within the last decades demonstrating that low-intensity strength training with external blood-flow restriction (BFR) induces similar effects compared to resistance training with heavier loads (65 + % 1RM). For instance, significant improvements in muscle mass, strength, and fatigue resistance were reported (Loenneke and Pujol, 2009; Luebbers et al., 2019; Pignanelli et al., 2020). BFR-training is characterized by a short-term, external restriction of the blood flow of the exercising muscles during the training session (Loenneke, 2011). This restriction is usually induced by inflatable cuffs or elastic bands which are wrapped around the proximal parts of the upper or lower extremities and typically applied with an individual cuff pressure ranging between 50 and 200 mmHg (Loenneke et al., 2012). Recent studies indicate that wider cuffs and higher arterial occlusion pressure might be more beneficial for improvements in power output and bar velocity due to increased mechanical compression (Gepfert et al., 2020; Wilk et al., 2020b,c). During continuous BFR (cBFR)-training, the cuffs are usually inflated before the exercise and deflated once the exercise is carried out. Since the cuffs are kept inflated throughout the entire exercise, the applied pressure alters the blood flow through a reduction in arterial influx and a concomitant block of venous return. This creates a state of increased metabolic stress for the exercising muscles due to the inability to remove the accumulated metabolites through the venous system (Pearson and Hussain, 2015).

However, high rates of perceived exertion and discomfort have been reported for cBFR training (Wernbom et al., 2006; Neto et al., 2018). One possible way of reducing discomfort might be the implementation of intermittent BFR-training which (1) is commonly characterized by deflated cuffs during the resting intervals (Yasuda et al., 2013; Freitas et al., 2019) and (2) has produced significant adaptations regarding hypertrophy (Freitas, 2020) and peak bar velocity (Wilk et al., 2020d). Although this approach leads to a reduced total time under BFR, deflating the cuffs during resting intervals do not seem to alter perceptual responses (Freitas et al., 2019). Alternatively, another way of BFR-training could be applied by solely inflating the cuffs during resting intervals (rBFR). Briefly, contractions of 15–20% of the maximal voluntary contraction can cause intramuscular pressure that impairs arterial blood flow (De Ruiter et al., 2007). This natural occurring ischemia probably maintains metabolic stress and hypoxia when supplemented with external restriction during inter-set rest. Termed as “metabolic freeze,” this has already been theorized by Okita et al. (2019). Using a cross-sectional approach, the authors reported that the resting BFR protocol lead to significant lower rates of perceived exertion (RPE). A recent work by Wilk et al. (2021) also showed enhanced bar velocity and power output in the bench press after ischemic conditioning during resting intervals compared to a control group without ischemic conditioning. Those results indicate possible benefits of resting BFR in terms of explosiveness and strength development in professional athletes.

Therefore, the main aim of this study was to find out whether (1) rBFR reveals lower rates of discomfort and perceived exertion than cBFR while (2) inducing comparable gains in hypertrophy, maximum strength, and fatigue resistance. Referring to what has been reported for strength development (Wilk et al., 2021), we hypothesize that rBFR induces similar hypertrophy, maximum strength, and fatigue resistance adaptations compared to cBFR.

## Materials And Methods

### Participants

**Figure 1:**
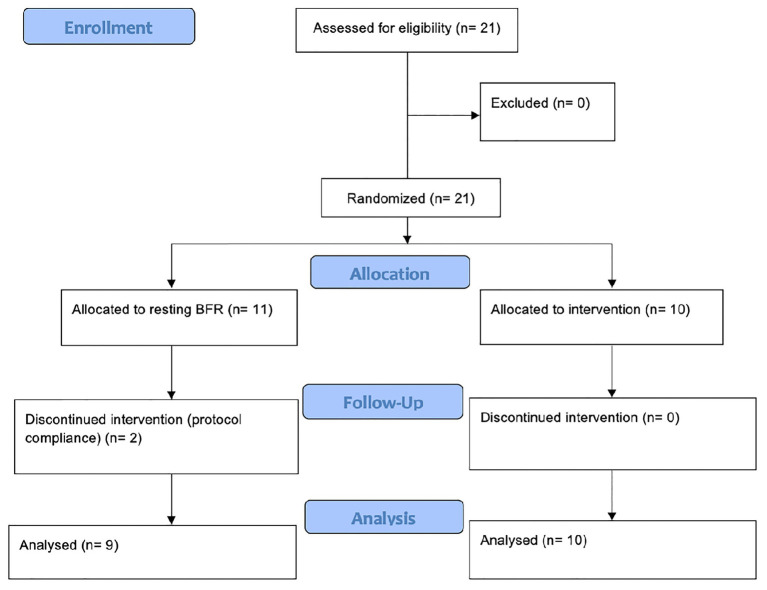
Flow chart of study conduction (Moher et al., 2001).

Since data are lacking to adequately calculate an effect size regarding an rBFR protocol, no *a priori* power analysis was conducted. Instead, we agreed to recruit 21 recreationally trained participants in order to account for dropouts while having enough power to examine possible between-group differences. As there were two dropouts due to lacking protocol compliance, only 19 participants (all male, 22.8 ± 1.8 years, 78.9 ± 3.71 kg, 179.6 ± 4.3 cm) were included in the data analysis (Figure 1). All participants were randomly assigned to two groups (continuous BFR = cBFR, *n* = 10 and resting BFR = rBFR, *n* = 9) using an online tool (“Random Team Generator”).^1^ While the cBFR group had their cuffs inflated throughout all load sets and rests, the rBFR group applied BFR only during the rest periods.

Participants were only included when they (1) were healthy and physically active and (2) had experience in resistance training, quantified as a resistance training experience of 2–3 times a week with an average of 10 sets for each muscle group. Exclusion criteria included neurological or cardiovascular disorders and acute or lasting injuries on the lower extremities. Both groups were instructed to maintain their usual training and living habits over the period of the study. The participants were extensively elucidated about the risks of BFR training, data protection, privacy, the study goal, as well as the study conduction procedure. Particularly, interventional strains and requirements were highlighted. Every individual voluntarily agreed and provided written consent to participate in the study. The study was approved by the local ethics committee (ethics committee department 05, Goethe University, Frankfurt am Main, Germany, no.: 2018-69) and was conducted in accordance to the ethical standards set by the declaration of Helsinki. Furthermore, it was retrospectively registered at the German register for clinical trials (DRKS00023510/11.11.2020).

### Study Design and Training Protocol

The 6-week parallel research design investigated the effects of rBFR and cBFR on maximum strength, fatigue resistance, muscle thickness girth, pain sensation, and perceived exertion. The study was preceded by a familiarization phase to accustom the participants to the cuff pressure and the feeling of BFR during training. For familiarization, the participants completed the training protocol, but only with the weight of the sled (20 kg). On the same occasion, pretests on the 45° leg press were performed that consisted of a 1RM test and a fatigue test (AMRAP). Muscle thickness was determined by ultrasound and thigh circumference was measured with a tape. Pain sensation was assessed using visual analog scale and rating of perceived exertion was quantified using the Borg scale. The same tests were repeated after 3 (mid) and 6 weeks (post).

**Figure 2:**
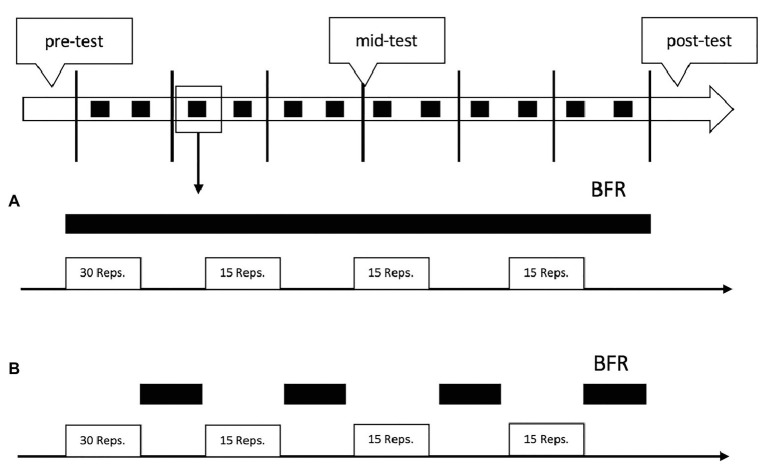
Schematic overview of the study design and training protocol. The black squares in the top figure represent the blood-flow restriction (BFR)-trainings. The black bars in **(A,B)** indicate the time under BFR during each training. **(A)** represents the training regimen of the continuous BFR (cBFR) group and **(B)** the training regimen of the resting BFR (rBFR) group.

Both groups had two supervised BFR-training sessions per week, as suggested by (Patterson et al., 2019). Each session lasted approximately 15 min, equating to an average workload of 30 min per week (Luebbers et al., 2019). We aimed for a between-training recovery of at least 1–2 days (Figure 2).

For both groups, each training session started with a warm-up set on the 45° leg press without BFR cuffs. Warm-up set weight was the same as during the BFR training (20% based on the 1RM as assessed in pre) and differed between 8 and 10 repetitions. The exercise protocol was based on a previous study by Madarame et al. (2013). Briefly, four sets were performed by both groups (30-15-15-15 repetitions) with 30 s of in-between-sets rest (Table 1). If the participants were able to complete two protocols with the predetermined weight the load was increased by 2.5 kg.

**Table 1:** Overview of training variables.

Protocol	Sets	Repetitions	1RM (%)	Rest interval (s)	Occlusion pressure (mmHg)
rBFR	4	30-15-15-15	20	30	183.33 ± 10.00
cBFR	4	30-15-15-15	20	30	186.00 ± 15.78

rBFR, resting BFR; cBFR, continuous BFR; 1RM, one repetition maximum. Data in means ± SD.

To account for contraction velocity, a metronome was used and set to 40 beats per minute as previously used in a similar protocol (Freitas et al., 2019). This resulted in contraction velocity of 1.5 s each concentric and eccentric phase. A hip-wide position was selected for all subjects and adjusted until 90° hip and knee flexion was achieved at the lowest position of the movement. Immediately after the completion of each set, all participants were instructed to move their legs into a more comfortable position so that both knees and the hip were relaxed.

### Blood-Flow Restriction

Inflatable cuffs (Signature Series BFR bands, Scottsdale, AZ, United States) were used for BFR-training which were 8 cm (3 inches) wide and 76 cm (30 inches) long. The cuffs were always positioned as proximally as possible to the thigh. Occlusion pressure was individually determined by Doppler ultrasound (Acuson X150, Siemens, Munich, Germany) with the participants lying in a supine position (Lixandrão et al., 2019). For blood-flow restriction, we determined the blood flow of the *arteria tibialis posterior* 5 cm proximal of the medial malleolus (Behringer et al., 2017). The cuffs were then inflated until no arterial pulse was visible or audible on the Doppler ultrasound (Husmann et al., 2018).

For the exercise protocol, 80% of the individual arterial occlusion pressure was prescribed as recommend by Lixandrão et al. (2015) in order to increase maximum strength and muscle thickness. In the rBFR group, the cuffs were inflated prior to the training protocol and immediately opened so that they were loose on the thigh but did not exert pressure. At the end of the set, both cuffs were closed as quickly as possible and reopened at the end of the interval. Since the cuffs lost some pressure after the first one or two sets, both hand pumps were constantly controlled, and the pressure readjusted if necessary. Contrarily, the cBFR group had continuous external BFR during working sets and resting intervals as described in various studies (Loenneke and Pujol, 2009; Yasuda et al., 2013; Freitas et al., 2019). An additional resting interval was added to both BFR protocols to increase comparability.

### Measurements

To avoid possible measuring interferences, all measures were obtained at least 48 h after a resistance training session (Yu et al., 2015; Yitzchaki et al., 2020).

### Maximal Strength

Since the loads of a 45° leg press can be very high, the test persons’ safety must be considered. We, therefore, estimated the 1RM from the 5-repetition maximum using Lombardi’s equation: 1RM = *R*^0.1^ × *W*, where *R* represents the repetitions and *W* the used weight. McNair et al. (2011) reported an excellent ICC of 0.97 for the Lombardi equation using the leg press. The study followed the recommendation of a recent review on strength testing (Grgic et al., 2020). Briefly, following a 5-min warm-up on the bicycle ergometer, the participants completed two submaximal warm-up sets on the leg press. Eight repetitions were performed with 50% of the estimated 1RM and three repetitions with 70% (Niewiadomski et al., 2008). There was a 3-min break between the sets to ensure sufficient regeneration. Thereafter, the resistance was increased until the participants were only able to perform five repetitions or less. The cadence of the RM tests was similar to contraction velocity of the training regimen, as emphasized by Wilk et al. (2020a). If the participants were not able to perform more than five repetitions, the test was stopped. Reliability measures showed excellent outcomes between pre, mid, and post (ICC = 0.903; CI: 0.808–0.958).

### Fatigue Resistance

During the fatigue test, participants were asked to perform as many repetitions as possible with 50% of the previously estimated 1RM on the 45° leg press. The fatigue test was stopped when the participants were unable to perform any further repetitions over the full range of motion (ROM) or when volitional failure was reached. A metronome was used and set to 40 beats per minute to provide the same standards as used in the regular exercise protocol.

### Muscle Thickness and Girth

Muscle thickness of the *M. rectus femoris* was measured using a B-mode transversal plane ultrasound (model Acuson X300, Siemens, Munich, Germany) using a 10 MHz linear-array probe (50 mm width). The participants were positioned as described above. For this study, muscle thickness of the *M. rectus femoris* was measured. The measuring point was halfway between the origin (*spina iliaca anterior inferior*) and the attachment (*tuberositas tibiae*) of the muscle. A water-resistant marking was placed at 50% of the segment length to increase repeatability (Rustani et al., 2019). The probe was then held over the marking with minimal pressure. A screenshot was taken from the ultrasound image and thickness was measured. Muscle thickness was defined as the distance between the lower margin of the upper fascia and the upper margin of the lower fascia of the *M. rectus femoris*. At each time point (pre-mid-post), three measurements were taken per participant and a mean value was determined from these three values. The ultrasound images were saved and used as a reference for mid- and post-tests to measure the muscle at the same location (Giles et al., 2017). In our lab, the ICC of muscle thickness using ultrasound has been found to be excellent (ICC = 0.963; CI: 0.913–0.985) in a sample size of *n* = 21, supporting its re-test reliability.

After measuring muscle thickness, girth was determined with the help of a tape. The participants were measured at the same point and in the same position as described for the ultrasound measurement (Doxey, 1987; Douciette, 1992).

### Perceived Exertion and Pain Sensation

Rates of perceived exertion was immediately assessed after the training using a BORG scale which ranged from 6 (no effort at all) to 20 (maximum effort). RPE scales are well-accepted tools to evaluate exertion in resistance training populations (Helms et al., 2020). Pain sensation was additionally determined using visual analog scale (VAS). The scale was 10 cm long (3.94 inches) and ranged from “no pain” to “worst pain imaginable” (Heller et al., 2016). Directly after the training, the participants were asked to mark the line as accurately as possible relating to their experienced pain. RPE and pain sensation were both measured with the BFR cuffs deflated. All participants were instructed about the scales and their proper utilization prior to each assessment.

### Data Presentation and Statistical Analysis

The data are presented as mean values ± SD. When adequate, effect sizes were reported. Boxplots and Shapiro-Wilk test were used to determine outliers and normal distribution of the data, respectively. A general linear two-way repeated measures ANOVA [time (3) × group (2)] with pairwise comparisons (Bonferroni correction) was performed separately for each dependent variable (SPSS version 24.0, Chicago, IL, United States). When a significant time × group interaction was revealed, simple main effects were examined separately using (a) a repeated-measures ANOVA (time) and (b) univariate ANCOVA covarying for t_1_ (group). Contrarily, if no significant interaction between group and time could be found, main effects for groups and time were interpreted as suggested by statistic-laerds (statistics.laerd.com, 2021). ANCOVA was performed additionally for the mid-post variables (RPE, VAS) using mid-values as the covariate. Statistical significance was set at *p* < 0.05.

## Results

Twelve BFR training sessions in total were performed by each participant during the course of the study with no differences between the groups (*p* > 0.05). During the intervention, there were no dropouts or injuries attributable to BFR. In sum, the four sets and the resting intervals resulted in 6 min training per individual session. With respect to the training protocols, the rBFR group increased their training weight from 59.29 ± 9.32 to 65.28 ± 7.75 kg (∆ +5.99 kg, 10.10%) over the course of the study. Contrarily, cBFR increased weights from 66.22 ± 17.91 to 68.35 ± 15.09 kg (∆ +2.13, 3.22%). No significant difference in training weight could be revealed for pre (*p* = 0.370), post (*p* = 0.845), or change (*p* = 0.845). Total tonnage was calculated for the training weight using the formula weight × repetitions × sets. While rBFR increased their total tonnage from 4446.43 ± 699.17 to 4895.83 ± 581.28 kg (∆ +449.4 kg, 10.11%), cBFR increased total tonnage in a similar extent [4966.67 ± 1343.62 to 5126.2 ± 1131.93 kg (∆ +159.58 kg, 3.21%)]. Since training weight was the only variable which changed throughout the study, total tonnage did not differ at any timepoint or between groups over time (*p* > 0.05). Descriptive are presented in Table 2.

**Table 2:** rBFR, resting BFR; cBFR, continuous BFR; 1RM, one repetition maximum; FR, fatigue resistance; MT, muscle thickness; VAS, visual analog scale; M, mean; SD, standard deviation; CI, confidence interval; *η*^2^, partial eta squared; d_z_, Cohen’s d.

		rBFR			cBFR		
		Pre	Mid	Post	Pre	Mid	Post
1RM (Kg)	M	314.78	316.00	341.44^*^^,^^†^	335.10	351.40^*^	366.30^*^^,^^†^
	SD	±42.25	±42.19	±32.26	±80.64	±83.84	±87.01
	CI (95%)	282.30–347.25	283.57–348.43	316.65–366.24	277.41–392.79	291.42–411.38	304.05–428.55
	*η*^2^			0.39			0.79
FR (reps.)	M	28.22	32.44^*^	38.33^*^^,^^†^	30.90	35.40^*^	38.80^*^^,^^†^
	SD	±7.77	±5.27	±8.11	±4.31	±5.17	±7.11
	CI	22.25–34.20	28.39–36.50	32.10–44.57	27.82–33.98	31.70–39.10	33.71–43.89
	*η*^2^			0.65			0.57
MT (mm)	M	22.93	23.20	23.62^*^^,^^†^	22.03	22.40^*^	22.91^*^^,^^†^
	SD	±2.87	±2.80	±2.94	±1.59	±1.51	±1.53
	CI	20.72–25.14	21.05–25.35	21.36–25.88	20.89–23.17	21.32–23.48	21.81–24.01
	*η*^2^			0.80			0.76
Girth (mm)	M	54.62	54.89	55.02	55.10	55.45	55.89^*^
	SD	±2.51	±2.42	±2.41	±3.36	±3.10	±3.37
	CI	52.69–56.55	53.03–56.75	53.17–56.87	52.70–57.50	53.23–57.67	53.48–58.30
	*η*^2^			0.15			0.35
BORG (RPE)	M		13.22	13.89		14.90^#^	14.00^†^
	SD		±1.30	±1.36		±1.10	±1.25
	CI		12.22–14.22	12.84–14.94		14.11–15.69	13.11–14.89
	d_z_			0.47			−0.82
VAS (mm)	M		28.33	28.00		48.50^#^	44.20
	SD		±14.54	±10.59		±13.92	±10.30
	CI		17.15–39.51	19.86–36.14		38.54–58.46	36.83–51.57
	d_z_			−0.04			−0.53

* Significant difference to pre-value.

# Significant difference between groups.

† Significant difference between mid and post.

### Muscle Strength

**Figure 3:**
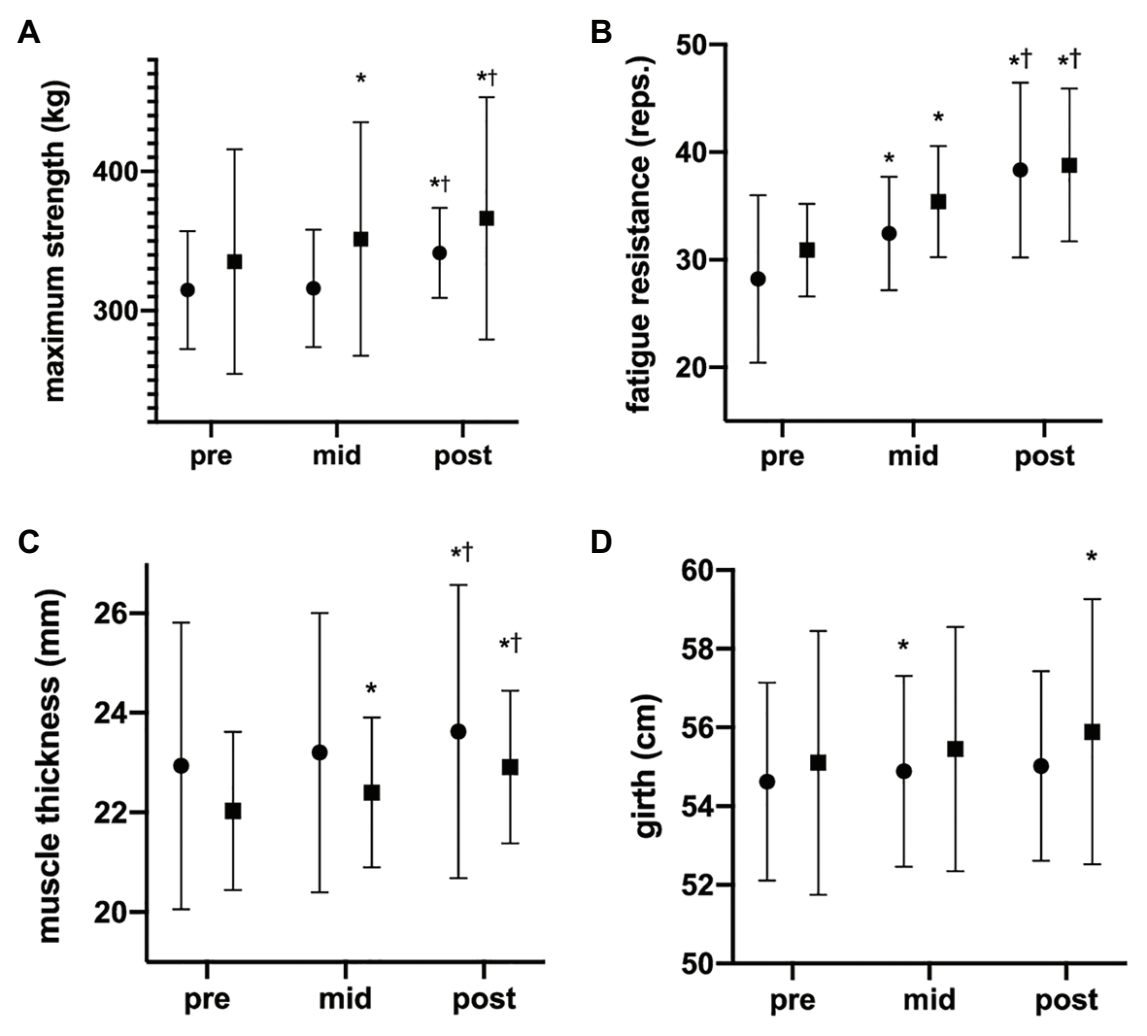
Development of maximum strength, fatigue resistance, muscle thickness, and girth. The data are presented as mean values ± SD. The graphs show **(A)** 1RM, **(B)** fatigue resistance, **(C)** muscle thickness, and **(D)** girth changes during the 6 weeks of the study (pre = after 0 weeks, mid = after 3 weeks, and post = after 6 weeks). [*] = statistically significant difference to pre-value; • = rBFR, ■ = cBFR. ^†^Significant difference between mid and post.

Both rBFR and cBFR increased muscle strength from 314.8 ± 42.3 to 341.7 ± 32.2 kg (∆ +26.9 kg, 8.5%) from 335.1 to 366.3 kg (∆ +31.2 kg, 9.3%), respectively (Figure 3). As the Box’s test revealed statistical significance, we separately assessed change over time for each group (ANOVA with repeated measures) as well as differences between groups for each time point (univariate ANOVA). Simple main effect time revealed a significant difference in maximum strength for rBFR *F*(1.141, 9.128) = 5.076, *p* = 0.047 between pre and post and for cBFR F(1.139, 10.248 = 32.904 = *p* < 0.001) between pre and mid, pre and post, as well as mid and post (*p* < 0.05). Contrarily, no significant differences between the groups could be seen for pre (*p* = 0.508), mid (*p* = 0.270), and post (*p* = 0.431), respectively.

### Fatigue Resistance

Both groups increased their fatigue resistance (rBFR: 28.2–38.3 ± 8.1 repetitions, ∆ +10.1 reps., 35.8%; cBFR: 30.9–38.8 ± 7.1 repetitions; ∆ +7.9 reps., 25.6%; Figure 3). Since the time × group interaction did not reveal significance (*p* = 0.545), main effects were interpreted. While both groups increased their fatigue resistance over time *F*(2,34) = 26.974, *p* < 0.001, we could not observe any between-group differences (*p* = 0.442). Pairwise comparisons revealed significant differences between pre- and mid (*p* = 0.002), mid and post (*p* = 0.002), and pre and post (*p* < 0.001).

### Muscle Thickness and Girth

Muscle thickness increased from 22.9 to 23.6 mm (∆ +0.7 mm, 3.05%) in the rBFR and from 22.0 to 22.9 mm (∆ +0.9 mm, 4.1%) in the cBFR group (Figure 3). The same trend could be observed in girth increasing from 54.6 to 55.0 cm (∆ +0.4 mm, 0.73%) in the rBFR and from 55.1 to 55.9 cm (∆ +0.8 cm, 1.45%) in cBFR group. There was no significant time × group interaction for muscle thickness *F*(1.390, 23.637) = 0.849, *p* = 0.402 or girth *F*(2, 34) = 0.670, *p* = 0.518. While muscle thickness (*p* = 0.970) and thigh circumference change (*p* = 0.638) did not differ between groups, both groups significantly increased muscle thickness *F*(1.390, 23.637) = 57.736, *p* < 0.001 and girth *F*(2,34) = 5.626, *p* = 0.008 over the study course. Pairwise comparisons revealed significant differences between pre- and mid, mid and post, and pre and post for muscle thickness (all *p* < 0.001) and pre and post for girth (*p* = 0.043).

### VAS and BORG Scale

**Figure 4:**
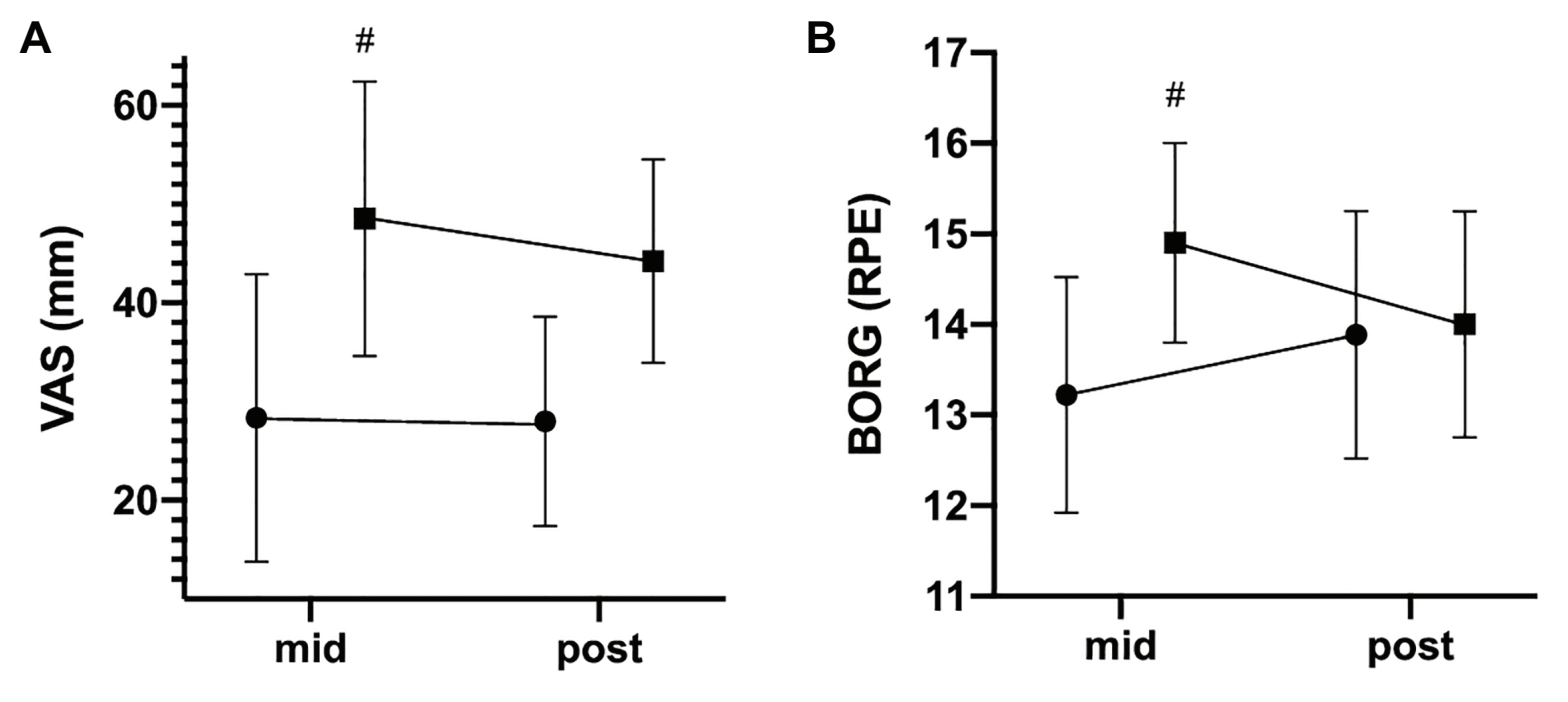
Development of perceived exertion and pain sensation. The data are presented as mean values ± SD. The graphs show the participants’ **(A)** pain sensation (VAS) and **(B)** perceived exertion on the Borg scale after 3 and 6 weeks. ^#^statistically significant difference between groups. • = rBFR, ■ = cBFR.

When analyzed for between-group differences, rBFR revealed a significant lower pain sensation (VAS) for mid *F*(1,17) = 9.530, *p* = 0.007 as well as perceived exertion for mid F(1,17) = 9.269, *p* = 0.007 when compared to cBFR (Figure 4). However, this between-group difference was no longer seen at post for either VAS (*p* = 0.266) or Borg scale (*p* = 0.855). Gain scores were additionally calculated to account for changes over time. However, no significant difference regarding delta change was found for either VAS (rBFR: ∆ −0.33 ± 8.47; cBFR: ∆ −4.33 ± 8.17, *p* = 0.266) or Borg scale (rBFR: ∆ +0.66 ± 1.41, cBFR: ∆ −0.90 ± 1.10, *p* = 0.249).

## Discussion

The main aims of this 6-week longitudinal study were to investigate whether rBFR (1) reveals lower rates of discomfort and perceived exertion compared to cBFR and (2) induces hypertrophy, strength gains, and fatigue resistance comparable to that of cBFR. To the authors’ best knowledge, the present study is the first to investigate the effects of rBFR training in a longitudinal fashion. All previous studies were cross-sectional and, therefore, limited to acute effects (Teixeira et al., 2018; Okita et al., 2019). In contrast to standard BFR training, the present study applied external restriction during the resting intervals (rBFR) with the aim of maximal metabolite accumulation.

With respect to discomfort, a significant lower pain perception while exercising (VAS) was revealed in the rBFR group at mid (*p* = 0.007), which was also seen in perceived exertion at mid (*p* = 0.007). These results are consistent with those of a previous study by Fitschen et al. (2014). In their study, the intermittent group recorded lower ratings of pain compared to the continuous protocol. Contrary, Freitas et al. (2019) did not find any differences between the intermittent and continuous BFR training groups in regard discomfort. Although using a training protocol similar to this study (Freitas et al., 2019), the intermittent BFR application differs to our work since we applied external restriction only during resting intervals thus limiting comparability. Since the rBFR group spend less time under BFR in total, we hypothesize that the lower levels of RPE and pain sensation result from reduced absolute metabolic stress, which is associated with higher levels of discomfort (Lagally et al., 2002). While the reasons explaining the differences between the intermittent protocols as well as the underlying mechanisms leading to lower discomfort in rBFR remain unclear, rBFR seems to be a viable alternative for athletes seeking to reduce high levels of discomfort during continuous BFR. Notably, between-group differences were no longer seen at post for both VAS (*p* = 0.266) and RPE (*p* = 0.855) indicating small to medium familiarization effects in cBFR (dz = −0.53). However, familiarization effects must be interpreted with great caution as delta change did not reveal significant group-differences probably attributed to high individual variance.

The present study revealed significant increases in maximum strength, fatigue resistance, muscle thickness, and girth with no differences between-groups. Maximum strength increased by ∆ +26.9 kg (8.5%) and ∆ +31.2 kg (9.3%) in the rBFR and cBFR group, respectively. Notably, effect sizes favor strength development in the cBFR condition (*η*^2^ = 0.79) compared to rBFR (*η*^2^ = 0.39) indicating that cBFR might be more effective in the long-term. However, rBFR still evoked a significant increase in maximum strength which is in line with previous research for continuous BFR-training (Yamanaka et al., 2012; Cook et al., 2014).

Fatigue tolerance also increased in both groups with the rBFR group improving by 10.1 repetitions (35.8%) and the cBFR group improving by +7.9 repetitions (25.6%). This is in line with previous findings (Fahs et al., 2015) reporting that cBFR can improve fatigue resistance in skeletal muscles by extending the resting levels of muscular glycogen and ATP. Briefly, BFR-training creates a hypoxic state in the trained muscle and leads to an accumulation of metabolites due to an increased ATP-hydrolysis outside the mitochondria (Robergs et al., 2004; Allen et al., 2008). Even though cBFR induces superior metabolic stress when compared to an intermittent BFR protocol relating to our findings (Suga et al., 2012), rBFR might be sufficient as well to alter local muscular environment.

In the context of structural changes, both groups were able to achieve significant improvements in muscle thickness and girth. Muscle thickness increased by ∆ + 0.7 mm (3.05%) in the rBFR group and ∆ + 0.9 mm (4.1%) in the cBFR group (both *p* < 0.001). Girth improved by ∆ + 0.4 cm (0.73%) in the rBFR group and ∆ + 0.8 cm (1.45%) in the cBFR group (*p* = 0.008). Since both groups significantly improved muscle thickness and girth, both continuous and resting BFR seem to elicit region-specific muscle hypertrophy in recreationally trained males. Various studies have reported significant muscle hypertrophy following BFR-training, mainly explained through high levels of metabolic stress (Yasuda et al., 2011; Conceição et al., 2019; Ramis et al., 2020). Current theory lists metabolite-induced fatigue and cell swelling and as the most likely mechanisms underpinning BFR-training benefits. Relating to the results reported in this study, inflating the cuffs only during resting intervals (rBFR) might already induce a sufficient accumulation of metabolic stress. This is in line with recent research reporting no physiological differences between intermittent and continuous BFR. In a previous study, Okita et al. (2019) compared several low-intensity BFR protocols (20 and 40% 1RM) including intermittent BFR (iBFR; BFR during exercise) and resting BFR (rBFR; BFR during resting intervals) protocols. Contrary to Freitas et al. (2020), they concluded that blood flow might be sufficiently restored when the cuffs are deflated during resting intervals finally leading to hampered muscle hypertrophy. In this context, rBFR might be a more effective strategy to maximize hypertrophy since inflating the cuffs during rest might create a metabolic freeze in the exercising muscles as proposed by Okita et al. (2019). In particular, metabolic stress might be maintained due to the inability of metabolite clearance during resting intervals.

## Conclusion

In conclusion, our findings indicate that BFR during resting intervals only (rBFR) might serve as an effective alternative to cBFR-training regarding maximum strength, fatigue resistance, and muscle thickness in recreationally trained males. At the same time, participants reported significantly lower discomfort during rBFR compared to cBFR. This could implement rBFR as an effective BFR alternative in professional sports and rehabilitation. Nevertheless, it should be noted that the obtained results refer only to the training regimen used as in this study, which does not have to translate into the same results. Therefore, further research is warranted with those specific populations.

## Limitations

The present study is not free of limitations. All Participants of this study were asked to continue their normal training routine in order to minimize bias in outcomes. Since we did not explicitly account for volume differences between the groups, the obtained results cannot exclusively be attributed to the BFR training. Another limitation of this study is the lack of a control group without BFR. This would have allowed to better evaluate the BFR induced effects of each training protocols. Also, no women were included due to the differences in hypertrophic response to resistance training compared to men.

## Data Availability Statement

The raw data supporting the conclusions of this article will be made available by the authors, without undue reservation.

## Ethics Statement

The present study involving human participants was reviewed and approved by Ethics Committee Department 05, Goethe University, Frankfurt am Main, Germany. The patients/participants provided their written informed consent to participate in this study.

## Author Contributions

All authors contributed to designing the study, analyzing and interpreting the data, and writing and proofreading the manuscript. All authors also approved the content of the manuscript’s final version.

### Conflict of Interest

The authors declare that the research was conducted in the absence of any commercial or financial relationships that could be construed as a potential conflict of interest.

## Footnotes

^1^www.jamestease.co.uk/team-generator
